# Douyin as a source of information and education on humeral supracondylar fracture of children during the COVID-19 pandemic in Chinese Mainland: An observational study

**DOI:** 10.1097/MD.0000000000034104

**Published:** 2023-06-23

**Authors:** Zhitao Zhu, Yan Zheng, Dongsheng Zhu

**Affiliations:** a Department of Radiology, The Second People’s Hospital of Lianyungang, Lianyungang, China; b Department of Pediatric Surgery, The First People’s Hospital of Lianyungang, Affiliated to Xuzhou Medical University, Lianyungang, China.

**Keywords:** quality of information, children, COVID-19, Douyin, humeral supracondylar fracture

## Abstract

We aimed to investigate whether Douyin videos on pediatric humeral supracondylar fractures could be a useful source during the COVID-19 pandemic. A search was conducted using the term “humeral supracondylar fracture of children” on Douyin. The top 100 videos were selected based on view count. 74 was the final analysis, after excluding 26 videos for various reasons. First, the videos were classified into medical and the non-medical groups based on authorship. The medical team videos were about explanations or detailed surgical procedures directly related to child’s fracture. There were also non-medical videos, mostly about personal experiences and other things. The videos were then also divided into 2 groups abased on the year of COVID-19 pandemic. The number of views, content type, video duration and number of likes about the video were analyzed. Among the 74 videos included in this study, had a total of 19,647,988 views (median 205,129, range 7874–1,495,004). Compared to the medical group, the non-medical group had more views (*P* = .004), likes (*P* = .000), view ratio (*P* = .019), and video power index (*P* = .024). During the COVID-19 pandemic, views (*P* = .033), view ratio (*P* = .006), and video power index (*P* = .043) also increased. Douyin has been a valuable source of health information for patients during COVID-19 pandemic regarding the occurrence of humeral supracondylar fracture in children. Medical professionals and institutions should upload credible, informative videos and clear, high-quality, scientifically reviewed surgical footage of children with humeral supracondylar fracture. And the videos uploaded by medical professionals and filtered by Douyin’s staff appear to be necessary.

## 1. Introduction

**Figure 1.**
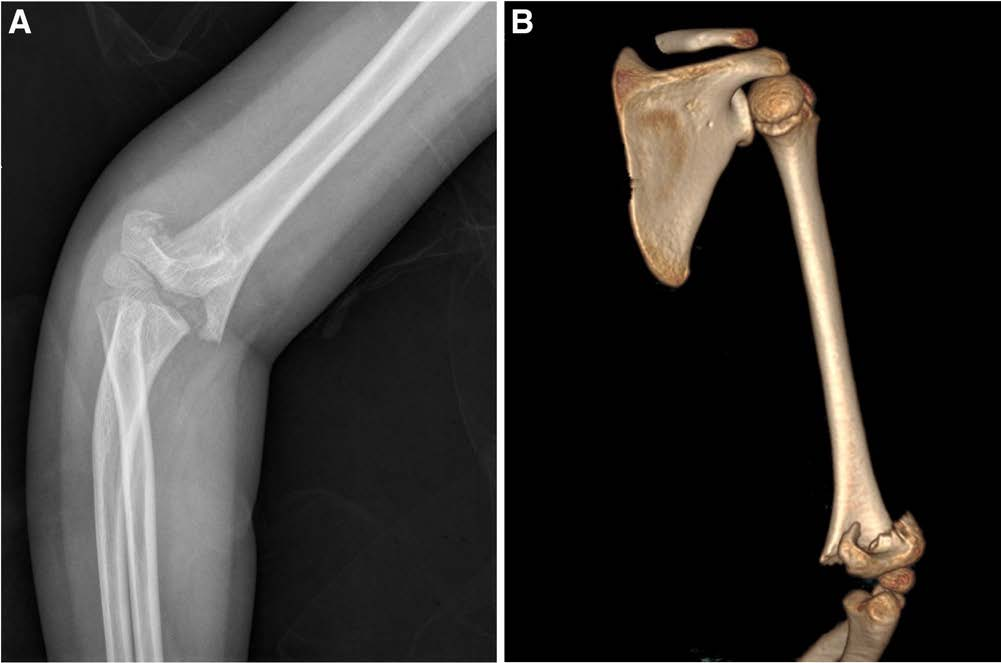
Imaging findings of supracondylar fractures of the humerus. A: X-ray; B: CT. CT = computed tomography.

Supracondylar humerus fractures (Fig. 1) are the most common childhood elbow fractures and accounts for about 10% of all pediatric fractures, according to previous reports.^[1,2]^ Cubitus varus is the most common deformity complication of pediatric supracondylar humerus fractures, with a reported incidence rate of between 3% and 57%.^[3]^ However, during the COVID-19 pandemic in the Chinese Mainland, patients tried to obtain relevant information from other sources due to difficulties in treatment. As previous reported, networks may provide access to a wide range of visual educational and online medical resources.^[4]^ As the anxiety levels of many guardians of children with pediatric supracondylar humerus fracture are increasing, it is important to use reliable Douyin videos to obtain appropriate information, especially during pandemic periods when medical treatment is inconvenient.

People are spending more time online due to social restrictions caused by COVID-19.^[5]^ The internet has already had a major impact on how the world communicates, with social media platforms facilitating mechanisms for instant information sharing.^[6]^ One of them is Douyin (Tik tok), a mobile app created in 2016 that has gained more than 800 million users in a few years and is the largest social online short video platform in China.^[7]^ However, a lack of professional information is often posted due to the lack of expertise of Douyin staff. In addition, there are still disputes about the reliability of the videos due to the lack of peer review.^[8]^ There has been no study on the information of supracondylar fracture of humerus in children from Douyin. Therefore, we analyzed the content of the videos related to pediatric supracondylar humerus fracture that were most played on Douyin and found out the characteristics of the videos related to pediatric supracondylar humerus fracture viewed by the public. In addition, we also evaluated the quality of these videos to determine whether they conveyed accurate and important information during the COVID-19 pandemic in the Chinese mainland.

## 2. Materials and methods

### 2.1. Ethics statement

The study was authorized by the ethics committee of the Second People’s Hospital of Lianyungang and followed the Declaration of Helsinki.

### 2.2. Search strategy

Using the term “supracondylar fracture of humerus,” a Douyin search was conducted on September 26, 2022. The following criteria were applied for inclusion: use of the Chinese language; videos related to supracondylar fracture of humerus; and adequate audiovisual quality. The exclusion criteria: use of languages other than Chinese; objectionable visual or audio; supracondylar fractures in adults; and duplicate videos.

**Figure 2.**
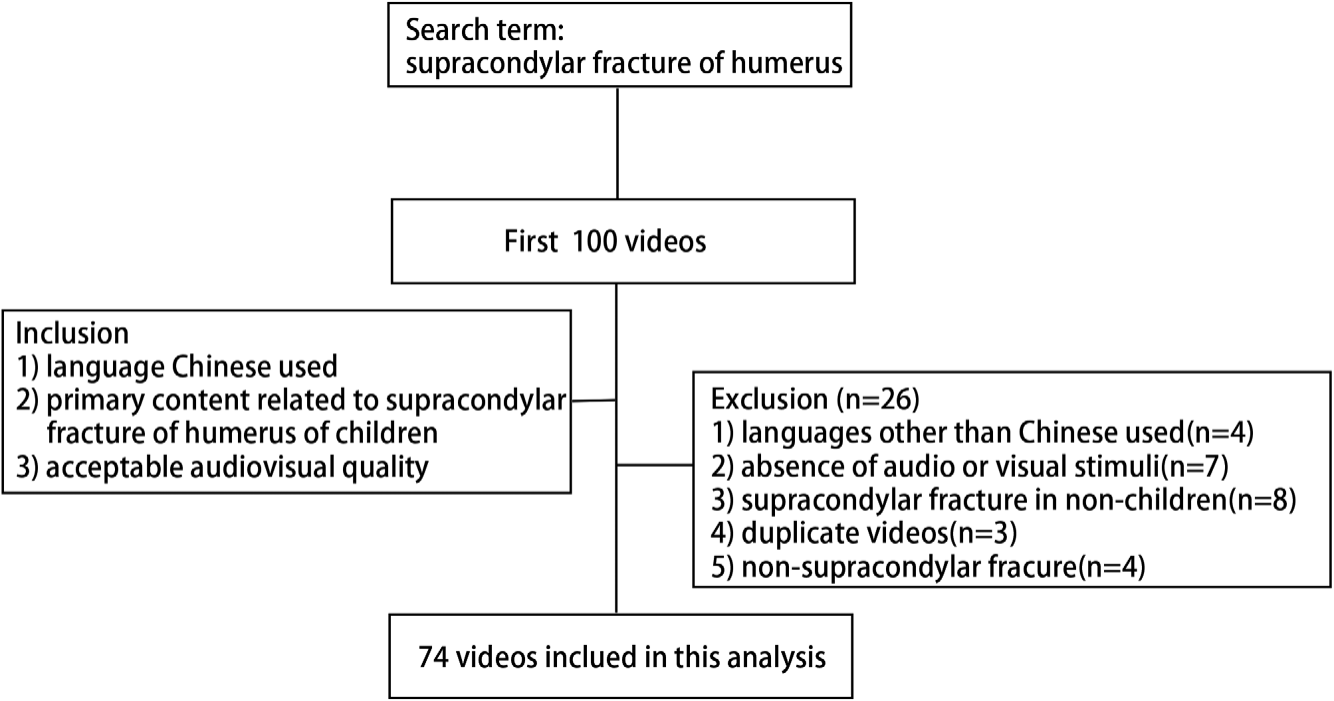
Methodology for selecting Douyin videos for the analysis.

The top 100 of the videos were chosen. The final analysis included 74 Douyin videos using the keywords “humeral supracondylar fracture of children” after 26 videos were excluded (languages other than Chinese used = 4, audio or visual unacceptable = 7, supracondylar fracture in non-children = 8, duplicate videos = 3, and non-supracondylar fracture = 4) (Fig. 2).

### 2.3. Video assessment

The general characteristics of each included video, including its type of content, number of views, duration in minutes, and the total number of “likes” as indicated by the “heart” icon, were all recorded. Based on the source of uploaded videos, we classified the videos into 4 categories: academics (authors were affiliated with a hospital), pediatric orthopedic surgeons, patients (guardians whose children have been diagnosed with humeral supracondylar fracture), commercial establishments, or auxiliary medical cares. Based on the content of the videos, they were categorized into 4 categories: Disease explanations (diagnosis, symptoms, and treatment associated with fracture of the humeral supracondylar in children); surgical procedures (explaining or showing surgical procedures); personal experiences (personal experiences related to humeral supracondylar fracture of children); and others (supplemental treatment options for children with humeral supracondylar fracture include nutrition and exercise).The videos were then divided into medical and non-medical groups. The former included videos uploaded by academic and pediatric orthopedic surgeons, and the latter included videos uploaded by patients, commercial establishments, and auxiliary medical cares (Table 1). At the end of 2019, COVID-19 was widespread in mainland China,^[9]^so we also divided the videos into 2 groups: pre-2019 and post-2019.

**Table 1:** Characteristics of videos related to the pediatric humeral supracondylar fracture on Douyin (n = 74).

Variables	Description	Value, n
Content		
Directly related		
Explanations of disease	Provide medical information related to supracondylar fracture of humerus of children, including medical treatment	24
Surgery videos	Show or explain detailed surgical procedures or techniques and processes	24
Indirectly related		
Personal experiences	Share personal experiences and feelings related to supracondylar fracture of humerus of children	10
Others	Complementary treatment options available for supracondylar fracture of humerus of children (e.g., nutrition and exercise)	16
Video authorship		
Medical		
Academic	Authors are affiliated with a hospital	19
Surgeon	Authors are a surgeon	20
Nonmedical		
Patient	Persons whose child have been diagnosed with supracondylar fracture of humerus of children and are currently undergoing treatment or have been treated	13
Commercial	Attention to a product or service	10
Paramedical	Allied health therapist, physiotherapist, or dietitian	12

Based on a previous study, we employed a validated scoring system: the global quality score to evaluate a the overall quality of healthcare videos (Table 2).^[10]^ The overall quality of the video was then assessed based on whether or not it contained important information about the humeral supracondylar fracture in children and how much scientific evidence was available.

**Table 2:** Global quality scale.

Rank	Quality	Score
Poor quality	Poor flow, most information missing, not helpful for patients	1
Generally poor	Some information given but of limited use to patients	2
Moderate quality	Some important information is adequately discussed	3
Good quality	Good flow, most relevant information is covered, useful for patients	4
Excellent quality	Excellent flow, useful for patients	5

Based on previous studies on YouTube, we used likes to assess the popularity of the videos, like ratio = likes/views*100, view ratio = number of views/days since upload, and video power index (VPI) = like ratio*view ratio/100.^[11,12]^ This was different from some previous studies, because the number of dislikes was not indicated in Douyin. And thus dislikes were not included in the video evaluation metric in this study.

### 2.4. Statistical analysis

The continuous variable video data were shown as median (range) values and compared using an unpaired test-test. The categorical variables were denoted as n (%) and were compared using Fisher’s exact test. The statistical analysis was conducted with Graphpad Prism 8, and the *P* value < .05 was considered statistically significant.

## 3. Results

A total of 19,647,988 (median 205,129, range 7874–1495,004) views were given to 74 videos related to pediatric humeral supracondylar fractures. The median time of the video was 4.60 (range 0.20–12.00) min and the average uploaded time of the videos was 622 (range 43–1947) days previously (Table 3). Analyzing the years of video uploads, the videos in this study were between 2017 and 2022, and 2021 was found to have the highest number of videos uploaded (Table 4).

**Table 3:** Descriptive features of videos related to humeral supracondylar fracture in children on Douyin (n = 74).

Variables	Median (range)
Views	205,129 (7146–1495,004)
Video length (min)	4.60 (0.20–12.00)
Time on Douyin (d)	622 (43–1947)
Likes (heart)	1467 (264–3408)
View ratio	336.21 (5.53–10,874.66)
Video power index	39.06 (1.13–3706.08)

**Table 4:** Number of videos included in the study by year of upload (n = 74).

Year	Uploaded videos, n
2017	7
2018	8
2019	12
2020	10
2021	21
2022	16

Based on video content and authorship, it was found that the most prevalent content of the videos was the explanation of humeral supracondylar fracture in children, which provided medical information related to the condition, including diagnosis, symptoms, and treatment of humeral supracondylar fracture in children (n = 24). For video authorship, it was found that videos uploaded by surgeons were the most (n = 20). And videos sharing the personal experiences and feelings of children with humeral supracondylar fracture were the fewest (n = 10). The medical and nonmedical groups uploaded 39 and 35 videos, respectively. After statistical analysis, it was found that the non-medical group had more views (*P* = .004), likes (*P* = .000), view ratios (*P* = .019), and VPI (*P* = .024), however, there was no significant difference in video length (*P* = .762), time on Douyin (*P* = .497), and score (*P* = .377) (Table 5). We further compared the videos uploaded before and after the end of 2019, as 2019 was the year in which COVID-19 was first detected in the Chinese Mainland and the world as a whole.^[9]^ Then, it was found that 27 and 47 videos were uploaded before and after the end of 2019 respectively. When analyzing the relationship between them, we found that the group after 2019 had more views (*P* = .033), view ratios (*P* = .006), and VPI (*P* = .043), however, video length (*P* = .451), likes (*P* = .421), and score (*P* = .555) were not significantly different (Table 6).

**Table 5:** Comparison according to the source of the videos.

Variables	Value, median (range)		*P* value
	Medical group (n = 39)	Non-medical group (n = 35)	
Views	76020 (7146–1495004)	362822 (7998–778158)	.004
Video length (min)	5.40 (0.40–12.00)	4.50 (0.20–11.60)	.762
Time on Douyin (d)	518 (55–1801)	665 (43–1947)	.479
Likes (heart)	1248 (264–2562)	1977 (792–3408)	.000
View ratio	226.35 (12.28–2814.29)	610.15 (5.53–10874.66)	.019
Video power index	27.84 (1.57–251.53)	86.86 (1.13–3706.18)	.024
Score	3 (1–5)	3 (1–5)	.377

**Table 6:** Comparison based on the time of upload of the videos to Douyin.

Variable	Value, median (range)		*P* value
	By the end of 2019 (n = 27)	After 2019 (n = 47)	
Views	118325 (7998–586,009)	260836 (7146–1495,004)	.033
Video length (min)	4.50 (0.20–10.90)	5.20 (0.30–12.00)	.451
Medical group video	44.44% (12/27)	57.45% (27/47)	.337
Likes (heart)	1467 (264–2639)	1468 (619–3408)	.421
View ratio	125.73 (5.53–396.30)	616.17 (42.65–10874.66)	.006
Video power index	45.94 (1.13–79.93)	544.91 (5.73–3706.08)	.043
Score	3 (1–5)	3 (1–5)	.555

## 4. Discussion

It was found that the most viewed video on Douyin about a child’s humeral supracondylar fracture was uploaded in 2020 by an account belonging to Dr Yao Jinghui, which provides information on the surgery. The second most viewed video on Douyin was also uploaded by Beijing Children’s Hospital in 2020. However, the highest VPI video was uploaded in 2021 by a parent of a child, which belongs to the non-medical group. Reflecting on the results, we found that the most viewed video does not necessarily have the highest VPI, since the VPI is an aggregate metric. In contrast to the medical group, VPI was found to be significantly higher in the non-medical group. Moreover, the medical group did not have significantly higher video scores than the non-medical group. Since the current research related to short videos and medical information is mainly related to YouTube, we compared out results to previous studies and found that our results are not similar to some previous studies Some scholars reviewed 50 videos on Youtube regarding meniscus and found that the videos uploaded by non-medical groups with information about meniscus tended to have lower video scores and often lower quality and reliability.^[13]^ In a study that analyzed YouTube videos of cardiopulmonary resuscitation by Turkey scholars, in their review, they reported a relationship between high VPI scores and low professional perspective.^[14]^ We believe that this may be related to the following reasons: With Douyin’s stricter audit system, low-quality videos may not be uploaded; and During the COVID-19 pandemic, normal ways of seeking medical care were affected, so internet-based medical care has risen in China. Non-medical units also paid great attention to online promotion, and they tend to upload high-quality videos.

It was found that the number of views, the view ratio, and VPI, which are indicators of the popularity of videos on Douyin, differ significantly between videos uploaded before 2019 and after 2019. We found that in terms of metrics that reflect the video popularity, the above metrics were significantly higher for video groups uploaded after 2019 than for those uploaded before 2019. Considering that the number of uploaded videos also increased after 2019, this could also be accounted for by the fact that Douyin became more popular during the COVID-19 pandemic and amateurs could easily access and produce content.

Several studies on YouTube have pointed out that the short video platform may be somewhat flawed in its function of transmitting medical information, as such information can be widely disseminated without filtering or scientific verification.^[15–17]^ At the same time, similar results were reported in the study of Douyin.^[18]^ In recent years, however, there has been a gradual increase in research on Douyin especially on its benefits.^[8,19]^ Because Douyin has a more rigorous audit system than YouTube, it is possible to use Douyin in the future to provide high-quality medical information in the field of pediatric humeral supracondylar fracture. Therefore, Douyin must strengthen the monitoring function of the active filtering process; in such a way that more useful medical knowledge can be provided during the COVID-19 pandemic.

It was found that the video of Douyin regarding humeral supracondylar fracture in children were provided a lot of useful information in this study. The results showed that videos with humeral supracondylar fracture of children are of high quality of information and Douyin is currently the appropriate source of such information for children with humeral supracondylar fracture. Medical professionals and medical institutions should upload credible and accurate videos and clear, high-quality, scientifically commented surgical clips to rigorously and quickly assess the quality of online video dissemination of pediatric humeral supracondylar fractures. In addition, it seems necessary for Douyin staff to use categories for advanced filtering. If necessary, even some medical related background staff may be employed.

There are some limitations to this study as well. We only analyzed 74 Douyin videos identified using the keyword “humeral supracondylar fracture of children.” The classification in this study was based on the number of views and included only Chinese videos, so there could be sampling bias. However, since there was no unified standard for evaluating videos, the scoring system was based on previous studies. Therefore, more validation is need to ensure that this evaluation method is applicable to accurately assess the video quality of pediatric humeral supracondylar fracture. In the future, we will further investigate the role of short videos in medical knowledge acquisition and dissemination, especially during the epidemics.

## Author contributions

**Conceptualization:** Zhitao Zhu, Yan Zheng, Dongsheng Zhu.

**Data curation:** Yan Zheng, Dongsheng Zhu.

**Formal analysis:** Yan Zheng, Dongsheng Zhu.

**Investigation:** Dongsheng Zhu.

**Methodology:** Dongsheng Zhu.

**Project administration:** Dongsheng Zhu.

**Resources:** Dongsheng Zhu.

**Software:** Dongsheng Zhu.

**Supervision:** Dongsheng Zhu.

**Validation:** Dongsheng Zhu.

**Visualization:** Zhitao Zhu, Dongsheng Zhu.

**Writing – original draft:** Dongsheng Zhu.

**Writing – review & editing:** Zhitao Zhu, Dongsheng Zhu.
